# Highly Sensitive p + n Metal Oxide Sensor Array for Low-Concentration Gas Detection

**DOI:** 10.3390/s18082710

**Published:** 2018-08-17

**Authors:** Jianghua Luo, Yishan Jiang, Feng Xiao, Xin Zhao, Zheng Xie

**Affiliations:** 1 Navy Submarine Academy, Qingdao 266199, China; 13061405315@sina.cn (J.L.); qdxuwx@126.com (F.X.); 13156239571@163.com (X.Z.); 2 State Key Laboratory of Chemical Resource Engineering, Beijing University of Chemical Technology, North Third Ring Road 15, Beijing 100029, China; zheng163xie@163.com

**Keywords:** metal oxide semiconductor, p + n sensor array, low concentration, ethanol and acetone sensing

## Abstract

Nowadays, despite the easy fabrication and low cost of metal oxide gas sensors, it is still challenging for them to detect gases at low concentrations. In this study, resistance-matched p-type Cu_2_O and n-type Ga-doped ZnO, as well as p-type CdO/LaFeO_3_ and n-type CdO/Sn-doped ZnO sensors were prepared and integrated into p + n sensor arrays to enhance their gas-sensing performance. The materials were characterized by scanning electron microscopy, transmittance electron microscopy, and X-ray diffractometry, and gas-sensing properties were measured using ethanol and acetone as probes. The results showed that compared with individual gas sensors, the response of the sensor array was greatly enhanced and similar to the gas response product of the p- and n-type gas sensors. Specifically, the highly sensitive CdO/LaFeO_3_ and CdO/Sn-ZnO sensor array had a high response of 21 to 1 ppm ethanol and 14 to 1 ppm acetone, with detection limits of <0.1 ppm. The results show the effect of sensor array integration by matching the two sensor resistances, facilitating the detection of gas at a low concentration.

## 1. Introduction

Nowadays, due to their easy fabrication and low cost, metal oxide (MOX) gas sensors have been widely investigated to be applied in inflammable gas alarms and toxic gas detection [1,2,3,4]. Although they are now extensively used to detect the leakage of inflammable gases such as H_2_, CH_4_, etc. in the range of 0.1–5%, their detection of low-concentration gases, such as air pollutants (e.g., formaldehyde in indoor air) and breath organics (e.g., acetone as a sensor for diabetes) at the ppb-ppm level, is still technologically challenging [5,6,7,8,9,10,11].

One major strategy to solve this problem is the development of highly sensitive MOX materials, such as stoichiometrically and morphologically tuned SnO_2_, ZnO, In_2_O_3_, and WO_3_ materials [12,13,14,15,16,17,18,19]. For example, Chen et al. reported that Fe_2_O_3_/SnO_2_ core–shell nanorods had a high response of 20 to 10 ppm ethanol [20], and Zhang et al. synthesized heterostructured ordered ZnO-Fe_3_O_4_ inverse opal materials for the highly sensitive detection of acetone with a response of 2 to 1 ppm acetone [13]. Meanwhile, there is another strategy to improve the gas response using a sensor array design. Generally, p- and n-type gas-sensing materials have reverse responses to the same gases, that is to say, resistance increases in p-type MOX with reductive gases (e.g., ethanol), while resistance decreases in n-type MOX [21]. Wang et al. firstly designed a p-type (Co_2_O_3_) and n-type (SnO_2_) gas sensor array in order to use the complementary effect of p- and n-type gas sensors [22]. Using this sensor array, a response of ~38 was obtained to 1000 ppm toluene [23], and humidity selectivity can also be achieved by a two-sensor array [5]. However, a limitation is that highly sensitive p-type MOX materials are usually scarce compared with n-type materials.

In this study, we synthesized novel p-type Cu_2_O and LaFeO_3_ materials for the integration of a p + n sensor array, combining them with typical n-type doped ZnO materials. The results showed that the response of the sensor array was similar to the response product of the p- and n-type gas sensors. Specifically, the CdO/LaFeO_3_ and CdO/Sn-doped ZnO sensor array had high responses of 21 and 14 to 1 ppm ethanol and acetone, respectively, with a low detection limit of <0.1 ppm. The synthesis of highly sensitive p-type materials and the resistance matching of the p- and n-type sensors are found to be the main aspects of sensor array designs.

## 2. Experimental Method

Cu_2_O, Ga-doped ZnO, and Sn-doped ZnO were synthesized using the precipitation method, and the LaFeO_3_ material was obtained by sol–gel self-ignition [24,25,26,27]. Typically, Cu_2_O is produced by the reduction of Cu (II) with glucose. Firstly, 1 mL of CuSO_4_ solution (0.68 M) was diluted in 17 mL of water, and 1 mL of sodium citrate (0.74 M) and 1 mL of Na_2_CO_3_ were then added to make a blue solution. This was then kept at 80 °C in a water bath and 1 mL of 1.4 M glucose was added. After reacting for 2 h, the precipitate was separated by centrifuge and rinsed with water and ethanol to obtain the Cu_2_O product. Ga- and Sn-doped ZnO were obtained by co-precipitation. Firstly, 50 mL of ZnSO_4_ and 2.2 mol % GaCl_3_ (or SnCl_2_) were added to 100 mL of NH_4_HCO_3_, and the precipitation was rinsed and calcined at 500 °C for 2 h to obtain the product. For LaFeO_3_, La(NO_3_)_3_, Fe(NO_3_)_3_, and citric acid were dissolved in water. Ammonia was added to the solution to maintain the pH at 6–7 and the solution turned into a sol in a 75 °C water bath. The sol was then dried in an oven at 130 °C to form a dry gel, which was then ignited and annealed at 600 °C for 2 h to obtain the product. CdO was decorated onto the materials by immersing the powders in a Cd(NO_3_)_2_ solution, which was then dried and calcined at 500 °C for 2 h.

**Figure 1:**
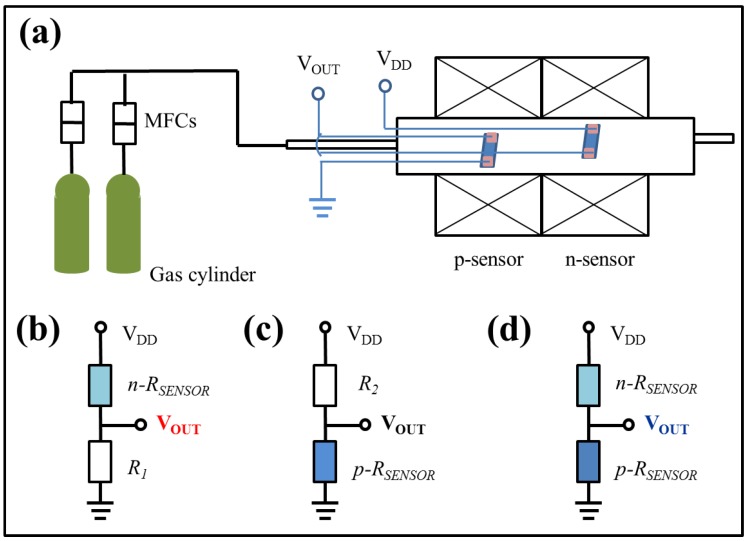
Schematics of the (**a**) gas sensor measurement system, (**b**) n-sensor, (**c**) p-sensor, and (**d**) p + n sensor array circuits.

The morphology and energy dispersive spectra (EDS) were observed using a scanning electron microscope (SEM, JEOL JSM-6700F, Japan, 15 kV, 10 μA) and a transmittance electron microscope (TEM, JEOL JEM-2010F, Tokyo, Japan, 200 kV, 100 µA). The crystal phase was identified by X-ray diffraction (XRD) on a Panalytical X-ray diffractometer with CuKα radiation of 0.154 nm (40 kV, 40 mA) and a high-resolution TEM (HRTEM). Gas-sensing performance was measured in a homemade gas sensor test instrument [28] as shown in Figure 1a. The sensing materials were drop-coated on Al_2_O_3_ substrates, which had two Pt wires attached on both ends by Ag paste. The two sensors were placed in a two-zone tube furnace to achieve the working temperature, and the gases to be detected were introduced into the tube furnace (diameter 40 mm) by a mass flow controller. The gas concentration of 0.1–1 ppm was controlled by diluting 5 ppm standard ethanol and acetone gases with synthetic air. Additionally, 1–5 ppm gas was obtained by diluting 50 ppm standard gas with synthetic gas. For example, 0.1 ppm ethanol was obtained by mixing 10 sccm 5 ppm ethanol gas with 490 sccm synthetic air. The voltage bias was 5 V, and the voltage (V_OUT_) on the load resistance was recorded for the n-type MOX sensor, while the voltage (V_OUT_) on the p-type MOX sensor was recorded for the p-type sensor and p + n sensor array as shown in Figure 1a–c. The response was defined as the resistance ratio of Ra/Rg for the n-type sensor and Rg/Ra for the p-type sensor to the reductive gases, where Ra and Rg are the sensor resistances in air and in detected gases, respectively, which are calculated from the measured voltage as follows: Ra and Rg = R (5/V_OUT_−1) [29].

## 3. Results and Discussion

**Figure 2:**
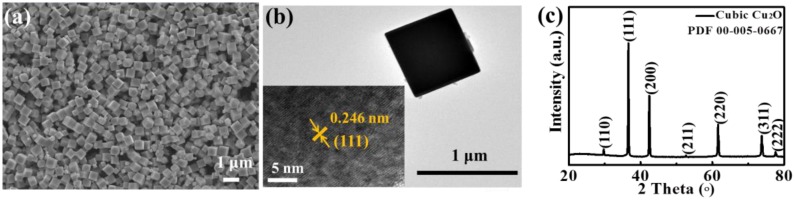
Characterization of p-type Cu_2_O sensing material: (**a**) SEM, (**b**) TEM and HRTEM (inset), and (**c**) XRD pattern.

The p-type Cu_2_O material was typically cubes, as shown in the SEM and TEM images in Figure 2a,b, with an edge length of ~0.5 µm. The HRTEM image in Figure 2b shows the typical (111) plane spacing of 0.246 nm in the cubic phase. The cubic phase was also identified by the XRD pattern in Figure 2c in good accordance with the standard card Powder Diffraction File (PDF) 00-005-0667. The cubic shape was attributed to the relatively low surface energy of the (100) surfaces as compared with others such as (111). This is in good agreement with the literature reporting that if surfactant (e.g., polyvinyl pyrrolidone) is used to stabilize the (111) plane, then octahedral shaped Cu_2_O would be obtained, exposing (111) planes, and if there is no surfactant, cubes would be produced, exposing (100) planes [30,31,32]. 

**Figure 3:**
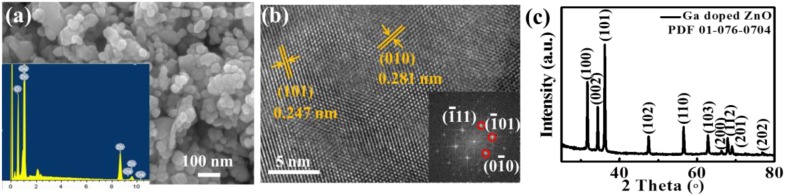
Characterization of n-type Ga-doped ZnO sensing material: (**a**) SEM and energy dispersive spectrum (EDS) (inset); (**b**) HRTEM and fast Fourier transmission (FFT) (inset); (**c**) XRD pattern.

Subsequently, another typical n-type sensing material, 2.2 mol % Ga-doped ZnO, was prepared and characterized as shown in Figure 3. As shown in the SEM image in Figure 3a, the Ga-ZnO nanoparticles had a diameter of 20–50 nm, and Ga was identified by the EDS spectrum as shown in the inset. The plane spacings in the HRTEM image and the fast Fourier transmission (FFT) in Figure 3b correspond well with the lattice of ZnO. There was no extra Ga_2_O_3_ phase identified by the XRD pattern besides the hexagonal ZnO phase (PDF 01-076-0704), as shown in Figure 3c, because the Ga atoms are doped into the ZnO lattice in order to tune the resistance. 

**Figure 4:**
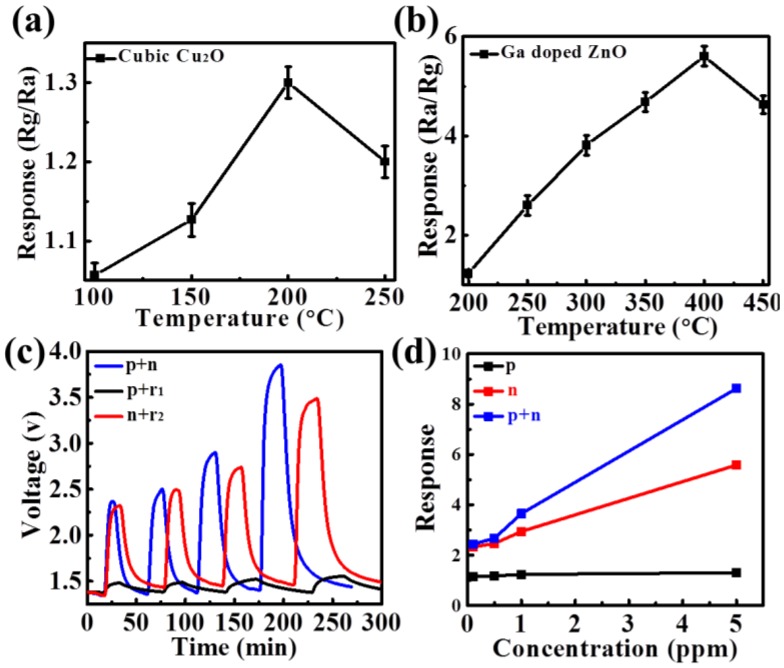
(**a**) Response of Cu_2_O p-sensor to 5 ppm ethanol at different working temperatures; (**b**) response of Ga-ZnO n-sensor to 5 ppm ethanol at different working temperatures; (**c**) responses of Ga-ZnO n-sensor, Cu_2_O p-sensor, and p + n sensor array to 5 ppm ethanol; and (**d**) relationship of Ga-ZnO n-sensor, Cu_2_O p-sensor, and p + n sensor array responses with ethanol concentrations.

The ethanol-sensing performance of Cu_2_O and Ga-ZnO were measured and calculated as shown in Figure 4a,b. Figure 4a shows that Cu_2_O had an optimal working temperature of 200 °C with a response of 1.3 to 5 ppm ethanol. This low working temperature ensures the high stability of the Cu_2_O sensor, as it is thermally oxidized into CuO at temperatures of >250 °C as reported in the literature [24,33]. Moreover, the resistance increasing in ethanol (Rg/Ra > 1) indicates the p-type conductivity of the Cu_2_O material. On the other side, Ga-ZnO showed a typical n-type sensing performance (Ra/Rg > 1 in ethanol) as shown in Figure 4b, with an optimal working temperature of 400 °C (response of 5.7 to 5 ppm ethanol). Subsequently, the p-type Cu_2_O and n-type Ga-ZnO sensors were integrated into a sensor array as shown in Figure 1c to measure their ethanol-sensing performance. As shown in Figure 4c, the dynamic response curves clearly show the enhanced response of the sensor array compared with the single sensors, with little influence on the response/recovery times. The response/recovery times were in the scale of several minutes, as the inlet gas needs a relatively long time of several minutes to achieve equilibrium in the large-volume quartz tube. Response versus ethanol concentration is plotted in Figure 4d, where a response of about 8.5 is obtained, similar to the response product of the Cu_2_O and Ga-ZnO sensors (1.3 × 5.7 = 7.4). 

**Figure 5:**
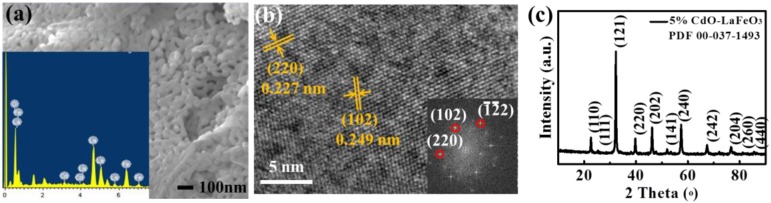
Characterizations of CdO-activated p-type LaFeO_3_: (**a**) SEM image and EDS spectrum (inset); (**b**) HRTEM and FFT (inset) and (**c**) XRD pattern.

It should be noted that although the sensor array had an enhanced response, this enhancement was limited by the relatively low response of the p-type Cu_2_O sensor. Therefore, we prepared another p-type sensing material, perovskite LaFeO_3_, which is activated by CdO decoration to enhance the response [21,34,35]. As shown in the SEM image in Figure 5a, the CdO/LaFeO_3_ material was composed of nanoparticles with a diameter of ~50 nm, with typical La, Fe, O, and Cd identified by the EDS spectrum in the inset. The HRTEM image and corresponding FFT in Figure 5b clearly show the typical perovskite (220) and (102) lattice spacings of LaFeO_3_. Furthermore, the XRD pattern shows the perovskite structure of LaFeO_3_ with no CdO peaks observed due to the relatively low dosage (5 mol %). 

**Figure 6:**
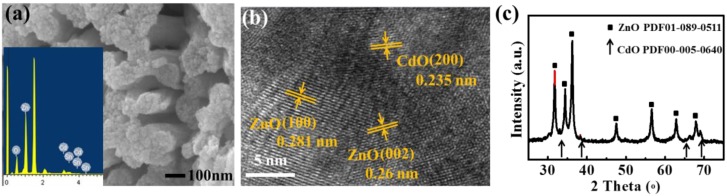
Characterizations of CdO-activated n-type Sn-doped ZnO: (**a**) SEM image and EDS spectrum (inset); (**b**) HRTEM and FFT (inset); and (**c**) XRD pattern.

However, this kind of LaFeO_3_ material has a relatively large resistance in the order of 10^9^ ohm, much higher than that of Cu_2_O and Ga-ZnO (10^6^ ohm), making it difficult to integrate the sensor array with Ga-ZnO. Therefore, another kind of n-type material, Sn-doped ZnO, was prepared and activated by 10 mol % CdO decoration [35] to enhance the sensitivity. As shown in Figure 6a, the CdO/Sn-ZnO material was also composed of nanoparticles with a diameter of 10–50 nm, and Cd, Sn, Zn, and O were all identified by the EDS spectrum in the inset. The lattice spacings in the HRTEM image in Figure 6b correspond well with the ZnO lattice. Importantly, the XRD pattern in Figure 6c indicates the hexagonal phase of the ZnO material, with extra CdO-related weak peaks identified. This is due to the relatively higher dosage of CdO (10 mol %) than that in CdO/LaFeO_3_. It is noticed that both materials, CdO-activated LaFeO_3_ and Sn-ZnO, have a porous structure as shown in Figure 5a and Figure 6a, which favors the gas diffusion and reaction and would thus enhance the gas response. 

**Figure 7:**
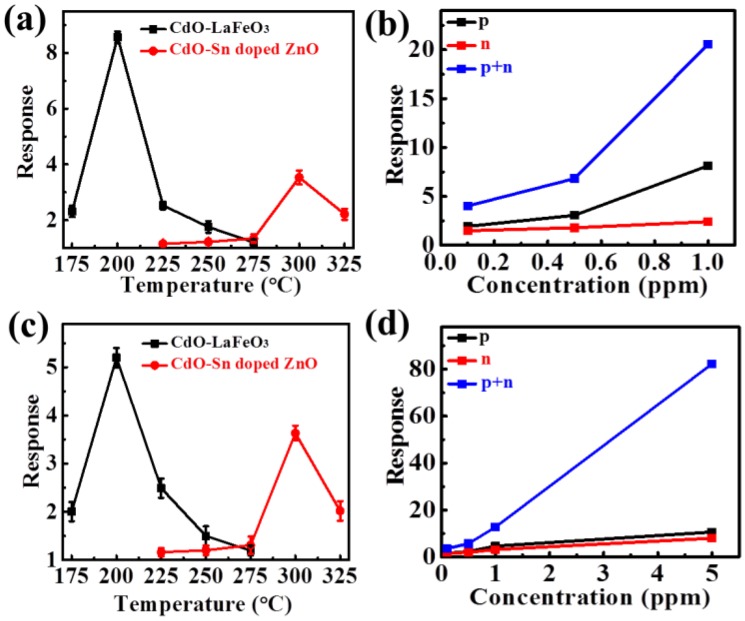
Responses of CdO-activated LaFeO_3_ and Sn-ZnO: (**a**,**b**) relationships of ethanol response with working temperature and concentration, respectively; (**c**,**d**) relationships of acetone response with working temperature and concentration, respectively.

Subsequently, these two sensors were integrated into a sensor array and the responses to typical ethanol and acetone gases are shown in Figure 7. The individual sensor responses of p-type CdO/LaFeO_3_ and n-type CdO/Sn-ZnO to 1 ppm ethanol were 8.5 and 3.5 at working temperatures of 200 and 300 °C, respectively, as shown in Figure 7a. The sensor array had an enhanced response of 21 to 1 ppm ethanol, similar to the product of 8.5 × 3.5 as shown in Figure 7b. The detection limit can be as low as 0.1 ppm for the sensor array with an obvious response of 4, showing the advantage of the sensor array in the detection of low-concentration gases. Normally, a response of 2–3 can be estimated as the detection limit; thus, the sensor array had a detection limit of <0.1 ppm. Furthermore, the responses to 1 ppm acetone were measured as shown in Figure 7c, where responses of 5.1 and 3.5 are observed for the CdO/LaFeO_3_ and CdO/Sn-ZnO sensors at optimal working temperatures of 200 and 300 °C, respectively. Thus, the sensor array had an enhanced response of 14 to 1 ppm acetone as shown in Figure 7d, with a low detection limit of <0.1 ppm. These are promising results for the use of this sensor array to detect traces of gas with concentrations at sub-ppm levels.

**Figure 8:**
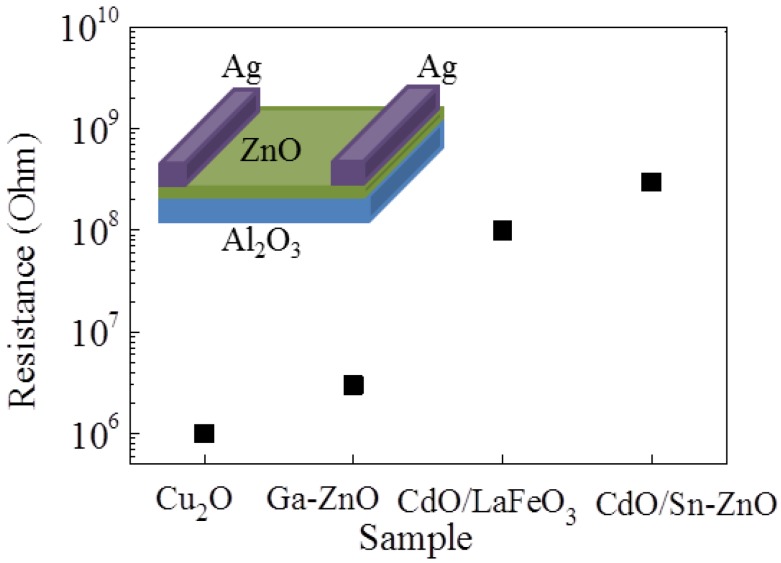
Gas sensor resistance selection rules for effective p + n sensor arrays.

However, there is an issue with the complex resistance matching of the sensors. As mentioned above, sensors with a respective resistance of 10^9^ and 10^6^ ohm cannot be integrated into an effective sensor array because there would not be any significant voltage change in the circuit, as depicted in Figure 1c. For example, the voltage output on the 10^6^-ohm sensor would be 0.005 V. A response of 10 would give an output voltage of only 0.05 V, which is too low to be effectively measured by the output circuits. The sensor resistances are plotted in Figure 8 to further illustrate this resistance matching problem. P-type Cu_2_O has a relatively low resistance, and that of n-type ZnO is orders of magnitude higher. Therefore, Ga was adopted as the effective dopant to reduce the resistance of n-type ZnO to a similar level to that of Cu_2_O. On the other hand, p-type LaFeO_3_ has a similar resistance to that of ZnO; thus, they can compose the sensor array. Additionally, the Sn dopant and the CdO activator were only used for enhancing the sensitivity of the material rather than tailoring the resistance. Therefore, all these results indicate the effective response enhancement of the well-tuned p + n sensor array for low-concentration gas detection.

## 4. Conclusions

P-type Cu_2_O and CdO/LaFeO_3_ as well as n-type Ga-doped ZnO and CdO/Sn-doped ZnO sensing materials were prepared, and effective p + n gas sensor arrays were designed and fabricated for the detection of low-concentration gas. The results showed that Cu_2_O and Ga-ZnO formed an effective sensor array with an enhanced response of 8.5 to 5 ppm ethanol. Moreover, the CdO/LaFeO_3_ and CdO/Sn-ZnO sensor array showed an enhanced response of 21 to 1 ppm ethanol and 14 to 1 ppm acetone with a low detection limit of <0.1 ppm. The high response of the sensor array is attributed to both the high response of the individual p- and n-type sensors and the multiplicity of the sensor array.
